# Supplementary material for: Detection of intracranial hypertension in children using optical coherence tomography: a systematic review protocol
Source: BMJ Open. 2020 Jul 6;10(7):e037833. doi: 10.1136/bmjopen-2020-037833 (PMC7342863; doi:10.1136/bmjopen-2020-037833)
Supplement: Supplementary data [file bmjopen-2020-037833supp001.pdf]

Appendices

Appendix 1: Search terms

Intracranial pressure, intracranial hypertension, papilloedema, optical coherence tomography

1. Intracranial pressure
2. ICP
3. Intracerebral pressure
4. Intracranial hypertension
5. Cerebral swelling
6. Cerebral oedema
7. Cerebral edema
8. Brain swelling
9. Brain oedema
10. Brain edema
11. Papilloedema
12. Papilledema
13. Optical coherence tomography
14. OCT

Search combination:

1 OR 2 OR 3 OR 4 OR 5 OR 6 OR 7 OR 8 OR 9 OR 10 OR 11 OR 12

AND

13 or 14

Appendix 2: Screening questions

Instructions for screeners: Tick the appropriate box per screening question. If "yes" at Stage 1, proceed to Stage 2; if "yes" at Stage 2, include. If "no" at any stage, exclude. If "unclear", proceed to next stage. If still "unclear" after Stage 2, then submit to third arbitrator for verdict.

Stage 1: Title Screening

Does the study represent Level IV evidence or above, i.e. case series, cohort studies, case-control studies, randomised controlled trials (RCTs) and systematic reviews?

Yes	
No	
Unclear	

Does the study pertain to the use of optical coherence tomography (OCT) to detect raised intracranial pressure (ICP) in children, defined as under 18 years of age?

Yes	
No	
Unclear	

Stage 2: Abstract screening

Does the study represent Level IV evidence or above, i.e. case series, cohort studies, case-control studies, randomised controlled trials (RCTs) and systematic reviews?

Yes	
No	
Unclear	

Does the study pertain to the use of optical coherence tomography (OCT) to detect raised intracranial pressure (ICP) in children, defined as under 18 years of age?

Yes	
No	
Unclear	

Stage 3: Full paper screening

Does the study represent Level IV evidence or above and pertain to the use of optical coherence tomography (OCT) to detect raised intracranial pressure (ICP) in children, defined as under 18 years of age?

Yes	
No	
Unclear	

Appendix 3: Data extraction tool, adapted from the Cochrane Collaboration

General Information

Study title:
Author(s):
Source:
Date of Extraction:

Methods

Aim of study	
Study design	
Inclusion criteria	
Exclusion criteria	
Method of recruitment	
Method of randomisation (if applicable)	
Blinding (if applicable)	
Study duration	
Ethics approval	

Participants

Population description	
Setting	
Total number randomised	
Mean age	
Male/Female %	

Intervention 1

Group name	
Number in group	
Description	
Intervention parameter 1	
Intervention parameter 2 (if applicable)	
Intervention parameter 3 (if applicable)	
Intervention parameter 4 (if applicable)	
Intervention parameter 5 (if applicable)	
Intervention parameter 6 (if applicable)	
Intervention parameter 7 (if applicable)	
Intervention parameter 8 (if applicable)	
Drop-outs from group	

Outcome 1

Outcome group name	
Time points measured	

Outcome definition	
Outcome parameter 1	
Outcome parameter 2 (if applicable)	
Outcome parameter 3 (if applicable)	
Outcome parameter 4 (if applicable)	
Outcome parameter 5 (if applicable)	
Outcome parameter 6 (if applicable)	
Outcome parameter 7 (if applicable)	
Outcome parameter 8 (if applicable)	
Person measuring	
Imputation of missing data	

Intervention 2 (if applicable)

Intervention group name	
Number in group	
Description	
Intervention parameter 1	
Intervention parameter 2 (if applicable)	
Intervention parameter 3 (if applicable)	
Intervention parameter 4 (if applicable)	
Intervention parameter 5 (if applicable)	
Intervention parameter 6 (if applicable)	
Intervention parameter 7 (if applicable)	
Intervention parameter 8 (if applicable)	
Drop-outs from group	

Outcome 2 (if applicable)

Outcome group name	
Time points measured	
Outcome definition	
Outcome parameter 1	
Outcome parameter 2 (if applicable)	
Outcome parameter 3 (if applicable)	
Outcome parameter 4 (if applicable)	
Outcome parameter 5 (if applicable)	
Outcome parameter 6 (if applicable)	
Outcome parameter 7 (if applicable)	
Outcome parameter 8 (if applicable)	
Person measuring	
Imputation of missing data	

Intervention 3 (if applicable)

Intervention group name	
Number in group	

Description	
Intervention parameter 1	
Intervention parameter 2 (if applicable)	
Intervention parameter 3 (if applicable)	
Intervention parameter 4 (if applicable)	
Intervention parameter 5 (if applicable)	
Intervention parameter 6 (if applicable)	
Intervention parameter 7 (if applicable)	
Intervention parameter 8 (if applicable)	
Drop-outs from group	

Outcome 3 (if applicable)

Outcome group name	
Time points measured	
Outcome definition	
Outcome parameter 1	
Outcome parameter 2 (if applicable)	
Outcome parameter 3 (if applicable)	
Outcome parameter 4 (if applicable)	
Outcome parameter 5 (if applicable)	
Outcome parameter 6 (if applicable)	
Outcome parameter 7 (if applicable)	
Outcome parameter 8 (if applicable)	
Person measuring	
Imputation of missing data	

Appendix 4: NIH Quality Assessment Tool for Observational Cohort and Cross-Sectional Studies

**Criteria**	**Yes**	**No**	**Other (CD, NR, NA)***
1. Was the research question or objective in this paper clearly stated?			
2. Was the study population clearly specified and defined?			
3. Was the participation rate of eligible persons at least 50%?			
4. Were all the subjects selected or recruited from the same or similar populations (including the same time period)? Were inclusion and exclusion criteria for being in the study prespecified and applied uniformly to all participants?			
5. Was a sample size justification, power description, or variance and effect estimates provided?			
6. For the analyses in this paper, were the exposure(s) of interest measured prior to the outcome(s) being measured?			
7. Was the timeframe sufficient so that one could reasonably expect to see an association between exposure and outcome if it existed?			
8. For exposures that can vary in amount or level, did the study examine different levels of the exposure as related to the outcome (e.g., categories of exposure, or exposure measured as continuous variable)?			
9. Were the exposure measures (independent variables) clearly defined, valid, reliable, and implemented consistently across all study participants?			
10. Was the exposure(s) assessed more than once over time?			
11. Were the outcome measures (dependent variables) clearly defined, valid, reliable, and implemented consistently across all study participants?			
12. Were the outcome assessors blinded to the exposure status of participants?			
13. Was loss to follow-up after baseline 20% or less?			
14. Were key potential confounding variables measured and adjusted statistically for their impact on the relationship between exposure(s) and outcome(s)?			

*CD, cannot determine; NA, not applicable; NR, not reported

**Quality Rating (Good, Fair, or Poor)**	
**Rater #1 initials:**	
**Rater #2 initials:**	
**Additional Comments (If POOR, please state why):**	
