# Supplementary material for: Detection of intracranial hypertension in children using optical coherence tomography: a systematic review protocol
Source: BMJ Open. 2020 Jul 6;10(7):e037833. doi: 10.1136/bmjopen-2020-037833 (PMC7342863; doi:10.1136/bmjopen-2020-037833)
Supplement: Author's manuscript [file bmjopen-2020-037833.draft_revisions.pdf]

BMJ Open

BMJ Open is committed to open peer review. As part of this commitment we make the peer review history of every article we publish publicly available.

When an article is published we post the peer reviewers' comments and the authors' responses online. We also post the versions of the paper that were used during peer review. These are the versions that the peer review comments apply to.

The versions of the paper that follow are the versions that were submitted during the peer review process. They are not the versions of record or the final published versions. They should not be cited or distributed as the published version of this manuscript.

BMJ Open is an open access journal and the full, final, typeset and author-corrected version of record of the manuscript is available on our site with no access controls, subscription charges or pay-per-view fees (http://bmjopen.bmj.com).

If you have any questions on BMJ Open's open peer review process please email info.bmjopen@bmj.com

BMJ Open

Detection of Intracranial Hypertension in Children using Optical Coherence Tomography: A Systematic Review Protocol

Journal:	*BMJ Open*
Manuscript ID	bmjopen-2020-037833
Article Type:	Protocol
Date Submitted by the Author:	18-Feb-2020
Complete List of Authors:	Rufai, Sohaib; Great Ormond Street Hospital For Children NHS Foundation Trust, Clinical and Academic Department of Ophthalmology; University of Leicester, University of Leicester Ulverscroft Eye Unit<br>Jeelani, Noor ul Owase; Great Ormond Street Hospital For Children NHS Foundation Trust, Department of Neurosurgery<br>McLean, Rebecca; University of Leicester, University of Leicester Ulverscroft Eye Unit
Keywords:	OPHTHALMOLOGY, Neuro-ophthalmology < OPHTHALMOLOGY, Paediatric ophthalmology < OPHTHALMOLOGY, PAEDIATRICS, NEUROSURGERY

SCHOLARONE™
Manuscripts

I, the Submitting Author has the right to grant and does grant on behalf of all authors of the Work (as defined in the below author licence), an exclusive licence and/or a non-exclusive licence for contributions from authors who are: i) UK Crown employees; ii) where BMJ has agreed a CC-BY licence shall apply, and/or iii) in accordance with the terms applicable for US Federal Government officers or employees acting as part of their official duties; on a worldwide, perpetual, irrevocable, royalty-free basis to BMJ Publishing Group Ltd ("BMJ") its licensees and where the relevant Journal is co-owned by BMJ to the co-owners of the Journal, to publish the Work in this journal and any other BMJ products and to exploit all rights, as set out in our licence.

The Submitting Author accepts and understands that any supply made under these terms is made by BMJ to the Submitting Author unless you are acting as an employee on behalf of your employer or a postgraduate student of an affiliated institution which is paying any applicable article publishing charge ("APC") for Open Access articles. Where the Submitting Author wishes to make the Work available on an Open Access basis (and intends to pay the relevant APC), the terms of reuse of such Open Access shall be governed by a Creative Commons licence – details of these licences and which Creative Commons licence will apply to this Work are set out in our licence referred to above.

Other than as permitted in any relevant BMJ Author's Self Archiving Policies, I confirm this Work has not been accepted for publication elsewhere, is not being considered for publication elsewhere and does not duplicate material already published. I confirm all authors consent to publication of this Work and authorise the granting of this licence.

For peer review only

Detection of Intracranial Hypertension in Children using Optical Coherence Tomography: A Systematic Review Protocol

Authors:

Dr Sohaib R. Rufai[1,2] (BMBS, MRes), Mr Noor ul Owase Jeelani[3,4] (MPhil, FRCS), Dr Rebecca J. McLean[1] (MSc, PhD)

Affiliations:

1. The University of Leicester Ulverscroft Eye Unit, Robert Kilpatrick Clinical Sciences Building, Leicester Royal Infirmary, Leicester, UK.
2. Clinical and Academic Department of Ophthalmology, Great Ormond Street Hospital for Children NHS Trust, London, UK Owase.
3. Department of Neurosurgery, Great Ormond Street Hospital for Children NHS Trust, London, UK
4. Developmental Biology & Cancer Department, UCL GOS Institute of Child Health, Faculty of Population Health Sciences, UCL, London, UK

Corresponding Author*

Dr Rebecca McLean (MSc, PhD)

The University of Leicester Ulverscroft Eye Unit, Robert Kilpatrick Clinical Sciences Building, PO Box 65, Leicester LE2 7LX, UK

E-mail: rjm19@leicester.ac.uk

Telephone: +44 (0)116 2523268

Fax: +44 (0)116 2231996

Funding: The Medical Research Council (London, UK) (grant no.: MR/N004566/1). SRR is funded by a National Institute for Health Research (NIHR) Doctoral Fellowship for this research project. This paper presents independent research funded by the National Institute for Health Research (NIHR) and MRC. The views expressed are those of the author(s) and not necessarily those of the MRC, the NHS, the NIHR or the Department of Health and Social Care.

Sponsor: University of Leicester

Role of sponsor or funder: The sponsor and funder had no role in developing this systematic review protocol.

Competing interests: The authors have no proprietary or commercial interest in any materials discussed in this study.

Keywords: Intracranial pressure, intracranial hypertension, optical coherence tomography, early recognition, screening, systematic review protocol

Word Count: 1760

For peer review only - http://bmjopen.bmj.com/site/about/guidelines.xhtml

Abstract

Introduction

Intracranial hypertension in children can have deleterious effects on the brain and vision. It is notoriously difficult to estimate intracranial pressure (ICP) in children and existing methods deliver sub-optimal diagnostic accuracy to be used as screening tools. Optical coherence tomography (OCT) may represent a valuable, non-invasive surrogate measure of ICP, as has been demonstrated in a number of associated conditions affecting adults. More recently, OCT has been employed within the paediatric age group. However, the role of OCT in detecting intracranial hypertension (ICH) in children has not been rigorously assessed in a systematic review for all relevant conditions. Here, we propose a systematic review protocol to examine the role of OCT in the detection of ICH in children.

Methods and analysis

Electronic searches in the Cochrane Central Register of Controlled Trials (CENTRAL), MEDLINE, Embase, Web of Science and PubMed will identify studies featuring OCT in detecting ICH in children. Two independent screeners will identify studies for inclusion using a screening questionnaire. Quality assessment will be performed using the National Institutes of Health (NIH) Quality Assessment Tool for Observational Cohort and Cross-Sectional Studies. The primary outcome measure is the sensitivity and specificity of OCT in detecting ICH in children. Secondary outcomes measures include conditions associated with ICH per study, direct ICP monitoring, sensitivity and specificity of other measures for ICP and OCT parameters used.

Ethics and dissemination: Ethical approval is not required for the proposed systematic review as no primary data will be collected. The findings will be disseminated through presentations at scientific meetings and peer-reviewed journal publication.

Trial registration number: International Prospective Register for Systematic Reviews (PROSPERO) number CRD42019154254

Strengths and limitations of this study

- The proposed study will be the first to examine the role of optical coherence tomography for all paediatric conditions associated with intracranial hypertension.
- This protocol provides transparency to the proposed systematic review methodology.
- This protocol reduces the possibility of duplication.
- The proposed methodology is recommended by the Cochrane Collaboration.

For peer review only - http://bmjopen.bmj.com/site/about/guidelines.xhtml

Introduction

Intracranial hypertension

Intracranial hypertension (ICH) was first described by Quincke in 1896 and remains a subject of major importance among the medical profession.[1] ICH can pose devastating consequences if untreated, including visual impairment, neurocognitive delay, disability and death.[2, 3] Subacute conditions in children can cause insidious elevation of intracranial pressure (ICP) which may go undetected before insult to the brain and vision has already begun.[2, 4] Hence, early detection of ICH is crucial in enabling timely medical intervention to prevent or limit the sequelae of ICH.

ICH affects between 0.63 and 0.71 per 100,000 children.[5, 6] ICH can be broadly classified as primary (or idiopathic) and secondary. Primary ICH is characterised by elevated ICP with no identifiable cause.[4, 7] Common risk factors for primary ICH include female gender, obesity and postpubertal status, although it is known to occur in children with a lack of predilection for gender and obesity.[4, 8-14] Secondary ICH describes elevated ICP that is a direct result of another condition, for example an expansive intracranial process, such as space occupying lesion or hydrocephalus, or a constricting skull pathology, such as the premature fusion of cranial sutures in craniosynostosis.[2-4]

Measurement of intracranial pressure

ICP measurement in children is notoriously difficult. The gold standard measure is direct intraparenchymal ICP measurement, which involves overnight admission to hospital and general anaesthesia. The risks of intraparenchymal ICP measurement include infection, haemorrhage, CSF leak and device failure.[15, 16] Furthermore, there is no universal agreement on timing, duration and frequency for accurate measurement or indeed agreement on the normal paediatric range of ICP – 11-15mmHg is widely considered normal, but Hayward *et al* argue that the upper limit could reasonably be raised to 20mmHg.[17, 18]

The ideal screening method for ICH should be sensitive, specific and child-friendly with the ability to record objective serial measurements over time. However, existing methods fail to satisfy all of these criteria. Visual evoked potentials (VEP) assess the amplitude and latency time of the averaged encephalographic response to visual stimulus. Axonal injury secondary to ICH is associated with reduction of amplitude or prolongation of the latency time.[2, 19] However, limitations of VEP include good the need for cooperation by the child, plus high variability in normal subjects.[20] Fundus examination can permit direct visualisation of papilloedema or optic atrophy, both of which are associated with ICH. A study by Tuite *et al* demonstrated that fundus examination had sensitivity of 100% in detecting ICH in children aged over 8 years, but only 22% for that in younger patients, excluding it as a suitable screening method. B-scan ocular ultrasound can be helpful in acute situations for detecting severe ICH, but possesses a sensitivity of only 11% when compared to fundoscopy.[21-24] Radiological signs, such as the “beaten copper” appearance of the cranium, universally demonstrate poor sensitivity for ICH.[25]

Optical coherence tomography

The optic nerve is primarily intracranial. Thus, ICH causes optic nerve head swelling, termed papilloedema, with secondary retinal changes. These changes can be measured objectively using optical coherence tomography (OCT) – a non-invasive imaging technique that can acquire ultra-high resolution, three-dimensional images of the back of the eye within seconds.[26] OCT has been used to describe the normal development of the optic nerve[27] and fovea[27, 28] in children. OCT has also been

For peer review only - http://bmjopen.bmj.com/site/about/guidelines.xhtml

utilised in a wide range of conditions within paediatric ophthalmology, including retinopathy of prematurity,[29] retinoblastoma,[30] nystagmus[31], albinism[32], achromatopsia[33], foveal hypoplasia,[34] optic nerve hypoplasia,[35] primary congenital glaucoma[36], microcephaly[37] and others. Furthermore, OCT has been successfully utilised as a diagnostic tool in a variety of conditions associated with ICH, including idiopathic intracranial hypertension, craniosynostosis and hydrocephalus.[38-44]

Thus, OCT may represent an ideal screening method for ICH in children owing to its quick acquisition time and non-invasive, child-friendly nature. However, this has not yet been rigorously assessed in a systematic review. Here, we propose to conduct the first systematic review examining the role of OCT in all conditions associated with ICH in children.

Objectives

The primary research objective of this proposed systematic review is to assess the sensitivity and specificity of OCT in detecting ICH in children.

The secondary research objectives are as follows:

- To assess which conditions associated with ICH have been successfully studied by OCT;
- To assess OCT success rate per condition per study;
- To identify surrogate measures of ICP and their associated sensitivity, specificity and success rates;
- To assess which ICP range is determined as normal per condition per study.

Methods

This protocol has adhered to the Preferred Reporting Items for Systematic review and Meta-Analysis Protocols (PRISMA-P) checklist.[45]

Eligibility criteria

The Population, Intervention, Comparison, Outcome and Study design (PICOS) strategy[46] has been employed to outline the eligibility criteria for this systematic review, as summarised in Table 1.

**PICOS strategy[46]**	**Inclusion criteria**	**Exclusion criteria**
P - Population	Studies of children, defined as being aged under 18 years, diagnosed with conditions associated with ICH.	i) Studies with participants over 18 years old;<br>ii) Studies not pertaining to the diagnosis of ICH.
I - Intervention	Studies employing OCT to detect ICH.	Any studies that do not include OCT.
C - Comparator	Absence of a comparator will not lead to exclusion of studies, as it may be unethical to deprive one arm of the study of OCT when it may lead to a better clinical outcome in such a dangerous situation of ICH.	N/A

For peer review only - http://bmjopen.bmj.com/site/about/guidelines.xhtml

O - Outcome	Sensitivity and specificity of any OCT measure(s), ± surrogate measure(s), for ICH.	Studies that do not report OCT measures.
S - Study design	All Level IV evidence and above, i.e. systematic reviews, randomised controlled trials (RCTs), case series and case-control studies, as defined by the Oxford Centre for Evidence-based Medicine.[47]	Level 5 evidence, i.e. expert opinion without critical appraisal.

Table 1: Eligibility criteria. Key: ICP = intracranial pressure; OCT = optical coherence tomography; PICOS = Population, Intervention, Comparison, Outcome and Study design.

Types of outcome measures

The main outcome measure will be sensitivity and specificity for OCT measures in detecting ICH. OCT metrics may include one or more of the following:

- Optic nerve parameters: cup depth, cup width, disc width and cup to disc ratio;
- Rim parameters: retinal nerve fibre layer (RNFL) thickness, rim volume, Bruch's membrane opening-minimum rim width (BMO-MRW), Bruch's membrane orientation, full peripapillary analysis;
- Retinal parameters: macular and perimacular retinal thickness, foveal pit width, depth and area, plus segmentation of all retinal layers.

The secondary outcome measures are as follows:

- Condition(s) associated with ICH per study;
- OCT success rate;
- Other surrogate estimates of ICP;
- Other OCT parameters not listed above;
- ICP range determined as normal.

Quality of life outcomes

Quality of life outcomes and patient satisfaction measured by surveys will be included where possible.

Adverse events

The process of OCT imaging cannot directly produce adverse events as it is a safe and non-invasive imaging method that uses low-coherence light.[48] It does not involve ionising radiation.

Follow-up

There will be no minimum follow-up period set for study inclusion. In many cases, the decision to employ rapid intervention (i.e. surgery) is made as soon as possible once ICH has been diagnosed. The stipulation of a minimum follow-up period would therefore risk losing valuable data from excluded studies.

For peer review only - http://bmjopen.bmj.com/site/about/guidelines.xhtml

Information sources

Record characteristics

There shall be no time restriction on records considered for this study. Unpublished records shall be considered. There shall be no language restrictions; all applicable non-English records will undergo professional translation from the University of Leicester Centre for Translation and Interpreting Studies.

Electronic searches

The following platforms shall be searched:

- Cochrane Central Register of Controlled Trials (CENTRAL) (including the Cochrane Eyes and Vision Group (CEVG) Trials Register)
- EMBASE Classic+Embase (1947 to present)
- Ovid MEDLINE(R) (1946 to present)
- Ovid MEDLINE In-Process and Other Non-Indexed Citations
- PubMed (1948 to present)
- Web of Science Core Collection (1970 to present)

Searching other resources

Unpublished studies identified by experts will be considered against our inclusion criteria. References of included studies will be searched and the authors contacted by e-mail or letter to identify further unpublished works where applicable. If the authors do not reply within 14 days, a reminder will be sent by e-mail or letter. If the authors do not reply to the reminder within 14 days, their study will be excluded.

Search strategy

Medical subject headings (MeSH) terms for 'intracranial hypertension' and 'optical coherence tomography' where entered into the above search platforms. Full details of search terms and strategy are included in Appendix 1.

Study records

Data management

EndNote X9 (Thomson Reuters, New York, NY) reference management software will be used for data management.

Selection of studies

Two independent screeners (SRR and RJM) shall follow a three-stage screening method. Appendix 2 outlines the screening questions and process. First, titles will be screened, followed by abstracts, followed by full papers. At each stage, studies shall be classified as 'include', 'exclude' or 'unclear', with those not excluded progressing to the next stage of screening. If any studies are classified as 'unclear' following the full paper screen, the study authors in question would be contacted by e-mail or letter for clarification. If the authors fail to reply within 14 days, a reminder will be sent. If the authors fail to reply to the reminder within 14 days, their study will be excluded. If there is any disagreement over which papers are selected for inclusion, the third arbitrator (NUOJ) shall make the final decision.

For peer review only - http://bmjopen.bmj.com/site/about/guidelines.xhtml

Data collection process

Our data extraction tool is included in Appendix 3, which is adapted from the Cochrane Collaboration.[49]

Data items, outcomes and prioritisation

Table 2 outlines the data items to be extracted. Extraction of primary outcome measures will be addressed as these will be used to address the primary research objective of this systematic review.

Data category	Data to be extracted
Study characteristics	• Author(s)<br>• Year<br>• Study design<br>• Study location<br>• Number of patients<br>• Mean age<br>• Age range
Primary outcome measures	• OCT Parameter(s)<br>○ Sensitivity (%)<br>○ Specificity (%)
Secondary outcome measures, where available	• Condition(s) studies;<br>• OCT success rate;<br>• Other surrogate estimates of ICP;<br>○ Sensitivity (%)<br>○ Specificity (%)<br>• Other OCT parameters not listed above;<br>• ICP range determined as normal.<br>• Quality of life outcomes<br>• Adverse events

Table 2: Data to be extracted. Key: ICP = intracranial pressure; OCT = optical coherence tomography.

For peer review only

Risk of bias in individual studies

The National Institutes of Health (NIH) Quality Assessment Tool for Observational Cohort and Cross-Sectional Studies,[50] included in Appendix 4, shall be used to assess risk of bias in the included studies.

Data analysis

Scoping searches suggest that mainly observational studies will be returned by our search strategy with few or no relevant RCTs. Weighted means for outcome measures will only be calculated if multiple RCTs are identified. Otherwise, we shall perform a qualitative review summarising the available evidence. A narrative synthesis will be conducted, examining the strengths, weaknesses, similarities and differences between included studies. Outcomes measures will be extracted and reported descriptively. Included studies will be evaluated based on the strength of the evidence.

For peer review only - http://bmjopen.bmj.com/site/about/guidelines.xhtml

Confidence in cumulative evidence

The Grades of Recommendation, Assessment, Development, and Evaluation (GRADE) approach[51] will be employed to evaluated the strength of the body of evidence identified in this study, where applicable.

Patient and public involvement

Involvement of patients and the public in the development of this systematic protocol is not applicable, as no patient recruitment will take place.

Dissemination

The findings of this systematic review shall be disseminated through presentations at scientific meetings, as well as peer-reviewed, open access journal publication.

Discussion

To the best of our knowledge, this is the first proposed systematic review to rigorously examine the role of OCT in detecting ICH in children. We have adhered to the PRISMA-P checklist[45] when developing this protocol. Two independent screeners shall conduct the search, with a third arbitrator available if required. Risk of bias will be assessed using the NIH Quality Assessment Tool for Observational Cohort and Cross-Sectional Studies.[50] This systematic review should help to clarify the role of OCT as a non-invasive screening method for ICH in paediatrics. Publication of this protocol should provide transparency to our proposed systematic review methodology as well as reduce the possibility of duplication.

Acknowledgements

We would like to thank Prof. Irene Gottlob and Dr. Frank Proudlock of the University of Leicester Ulverscroft Eye Unit, for their support and contributions towards this systematic review protocol.

Author Contributions

SRR: Concept, methodology, protocol writing, final approval.

NUOJ: Supervision, concept, critical revision, final approval.

RJM: Supervision, methodology, critical revision, final approval, guarantor of review.

For peer review only

For peer review only - http://bmjopen.bmj.com/site/about/guidelines.xhtml

References

1. Quincke, H., *Ueber Meningitis serosa und verwandte Zustände.* Deutsche Zeitschrift für Nervenheilkunde, 1896. **9**(3-4): p. 149-168.
2. Derderian, C. and J. Seaward, *Syndromic craniosynostosis.* Seminars in plastic surgery, 2012. **26**(2): p. 64-75.
3. Jordan, C.O. and S.C. Aylward, *Intracranial hypertension: a current review.* Curr Opin Pediatr, 2018. **30**(6): p. 764-774.
4. Aylward, S.C. and R.E. Reem, *Pediatric Intracranial Hypertension.* Pediatric Neurology, 2017. **66**: p. 32-43.
5. Matthews, Y.Y., et al., *Pseudotumor cerebri syndrome in childhood: incidence, clinical profile and risk factors in a national prospective population-based cohort study.* Arch Dis Child, 2017. **102**(8): p. 715-721.
6. Gillson, N., et al., *Incidence and Demographics of Pediatric Intracranial Hypertension.* Pediatr Neurol, 2017. **73**: p. 42-47.
7. Mollan, S.P., et al., *Idiopathic intracranial hypertension: consensus guidelines on management.* J Neurol Neurosurg Psychiatry, 2018. **89**(10): p. 1088-1100.
8. Radhakrishnan, K., et al., *Epidemiology of idiopathic intracranial hypertension: a prospective and case-control study.* J Neurol Sci, 1993. **116**(1): p. 18-28.
9. Raoof, N., et al., *The incidence and prevalence of idiopathic intracranial hypertension in Sheffield, UK.* Eur J Neurol, 2011. **18**(10): p. 1266-8.
10. Radhakrishnan, K., et al., *Idiopathic intracranial hypertension (pseudotumor cerebri). Descriptive epidemiology in Rochester, Minn, 1976 to 1990.* Arch Neurol, 1993. **50**(1): p. 78-80.
11. Cinciripini, G.S., S. Donahue, and M.S. Borchert, *Idiopathic intracranial hypertension in prepubertal pediatric patients: characteristics, treatment, and outcome.* Am J Ophthalmol, 1999. **127**(2): p. 178-82.
12. Kesler, A. and A. Fattal-Valevski, *Idiopathic intracranial hypertension in the pediatric population.* J Child Neurol, 2002. **17**(10): p. 745-8.
13. Babikian, P., J. Corbett, and W. Bell, *Idiopathic intracranial hypertension in children: the Iowa experience.* J Child Neurol, 1994. **9**(2): p. 144-9.
14. Phillips, P.H., M.X. Repka, and S.R. Lambert, *Pseudotumor cerebri in children.* J aapos, 1998. **2**(1): p. 33-8.
15. Zhang, X., et al., *Invasive and noninvasive means of measuring intracranial pressure: a review.* Physiol Meas, 2017. **38**(8): p. R143-r182.
16. Tamburrini, G., et al., *Intracranial pressure monitoring in children with single suture and complex craniosynostosis: a review.* Childs Nerv Syst, 2005. **21**(10): p. 913-21.
17. Renier, D., et al., *Intracranial pressure in craniostenosis.* J Neurosurg, 1982. **57**(3): p. 370-7.
18. Hayward, R., et al., *Connecting raised intracranial pressure and cognitive delay in craniosynostosis: many assumptions, little evidence.* J Neurosurg Pediatr, 2016. **18**(2): p. 242-50.
19. Thompson, D.A., et al., *Prevalence of abnormal pattern reversal visual evoked potentials in craniosynostosis.* Plast Reconstr Surg, 2006. **118**(1): p. 184-92.
20. Andersson, L., J. Sjölund, and J. Nilsson, *Flash visual evoked potentials are unreliable as markers of ICP due to high variability in normal subjects.* Acta Neurochirurgica, 2012. **154**(1): p. 121-127.
21. Dubourg, J., et al., *Ultrasonography of optic nerve sheath diameter for detection of raised intracranial pressure: a systematic review and meta-analysis.* Intensive Care Med, 2011. **37**(7): p. 1059-68.

For peer review only - http://bmjopen.bmj.com/site/about/guidelines.xhtml

22. Driessen, C., et al., *Nocturnal ultrasound measurements of optic nerve sheath diameter correlate with intracranial pressure in children with craniosynostosis.* Plast Reconstr Surg, 2012. **130**(3): p. 448e-51e.
23. Bolesch, S., et al., *Transcranial ultrasound to detect elevated intracranial pressure: comparison of septum pellucidum undulations and optic nerve sheath diameter.* Ultrasound Med Biol, 2015. **41**(5): p. 1233-40.
24. Driessen, C., et al., *Are ultrasonography measurements of optic nerve sheath diameter an alternative to funduscopy in children with syndromic craniosynostosis?* J Neurosurg Pediatr, 2011. **8**(3): p. 329-34.
25. Tuite, G.F., et al., *The beaten copper cranium: a correlation between intracranial pressure, cranial radiographs, and computed tomographic scans in children with craniosynostosis.* Neurosurgery, 1996. **39**(4): p. 691-9.
26. Lee, H., F.A. Proudlock, and I. Gottlob, *Pediatric Optical Coherence Tomography in Clinical Practice-Recent Progress.* Invest Ophthalmol Vis Sci, 2016. **57**(9): p. OCT69-79.
27. Patel, A., et al., *Optic Nerve Head Development in Healthy Infants and Children Using Handheld Spectral-Domain Optical Coherence Tomography.* Ophthalmology, 2016. **123**(10): p. 2147-57.
28. Lee, H., et al., *In Vivo Foveal Development Using Optical Coherence Tomography.* Invest Ophthalmol Vis Sci, 2015. **56**(8): p. 4537-45.
29. Anwar, S., et al., *POTENTIAL UTILITY OF FOVEAL MORPHOLOGY IN PRETERM INFANTS MEASURED USING HAND-HELD OPTICAL COHERENCE TOMOGRAPHY IN RETINOPATHY OF PREMATURITY SCREENING.* Retina (Philadelphia, Pa.), 2019: p. 10.1097/IAE.0000000000002622.
30. Soliman, S.E., et al., *Optical Coherence Tomography-Guided Decisions in Retinoblastoma Management.* Ophthalmology, 2017. **124**(6): p. 859-872.
31. Lee, H., F. Proudlock, and I. Gottlob, *Is handheld optical coherence tomography reliable in infants and young children with and without nystagmus?* Investigative ophthalmology & visual science, 2013. **54**(13): p. 8152-8159.
32. Mohammad, S., et al., *Characterization of Abnormal Optic Nerve Head Morphology in Albinism Using Optical Coherence Tomography.* Investigative ophthalmology & visual science, 2015. **56**(8): p. 4611-4618.
33. Thomas, M.G., et al., *High-resolution in vivo imaging in achromatopsia.* Ophthalmology, 2011. **118**(5): p. 882-887.
34. Rufai, S.R., et al., *Can Structural Grading of Foveal Hypoplasia Predict Future Vision in Infantile Nystagmus?: A Longitudinal Study.* Ophthalmology, 2019: p. S0161-6420(19)32212-2.
35. Pilat, A., et al., *High-Resolution Imaging of the Optic Nerve and Retina in Optic Nerve Hypoplasia.* Ophthalmology, 2015. **122**(7): p. 1330-1339.
36. Pilat, A.V., et al., *Assessment of the anterior segment of patients with primary congenital glaucoma using handheld optical coherence tomography.* Eye (London, England), 2019. **33**(8): p. 1232-1239.
37. Papageorgiou, E., et al., *Retinal and optic nerve changes in microcephaly: An optical coherence tomography study.* Neurology, 2018. **91**(6): p. e571-e585.
38. Optical Coherence Tomography Substudy, C. and N.I.I.H.S. Group, *Papilledema Outcomes from the Optical Coherence Tomography Substudy of the Idiopathic Intracranial Hypertension Treatment Trial.* Ophthalmology, 2015. **122**(9): p. 1939-45.e2.
39. Scott, C.J., et al., *Diagnosis and grading of papilledema in patients with raised intracranial pressure using optical coherence tomography vs clinical expert assessment using a clinical staging scale.* Archives of ophthalmology (Chicago, Ill. : 1960), 2010. **128**(6): p. 705-711.
40. Skau, M., et al., *Diagnostic value of optical coherence tomography for intracranial pressure in idiopathic intracranial hypertension.* Graefe's archive for clinical and experimental

For peer review only - http://bmjopen.bmj.com/site/about/guidelines.xhtml

ophthalmology = Albrecht von Graefes Archiv fur klinische und experimentelle Ophthalmologie, 2013. **251**(2): p. 567-574.
41. Anand, A., et al., *Optical coherence tomography of the optic nerve head detects acute changes in intracranial pressure.* Journal of clinical neuroscience : official journal of the Neurosurgical Society of Australasia, 2016. **29**: p. 73-76.
42. Patel, M.D., et al., *Methods for Quantifying Optic Disc Volume and Peripapillary Deflection Volume Using Radial Optical Coherence Tomography Scans and Association With Intracranial Pressure.* Frontiers in neurology, 2019. **10**: p. 798-798.
43. Swanson, J.W., et al., *Evaluation of Optical Coherence Tomography to Detect Elevated Intracranial Pressure in Children.* JAMA Ophthalmol, 2017. **135**(4): p. 320-328.
44. Swanson, J.W., et al., *Intracranial pressure patterns in children with craniosynostosis utilizing optical coherence tomography.* Child's nervous system : ChNS : official journal of the International Society for Pediatric Neurosurgery, 2019: p. 10.1007/s00381-019-04448-x.
45. Shamseer, L., et al., *Preferred reporting items for systematic review and meta-analysis protocols (PRISMA-P) 2015: elaboration and explanation.* BMJ : British Medical Journal, 2015. **349**: p. g7647.
46. Higgins, J.P. and S. Green, *Defining the review question and developing criteria for including studies*, in *Cochrane handbook for systematic reviews of interventions*. 2008, Wiley Online Library. p. 83.
47. CEBM. *Oxford Centre for Evidence-based Medicine – Levels of Evidence (March 2009)*. 24/10/2019]; Available from: https://www.cebm.net/2009/06/oxford-centre-evidence-based-medicine-levels-evidence-march-2009/.
48. Fujimoto, J.G., et al., *New technology for high-speed and high-resolution optical coherence tomography.* Ann N Y Acad Sci, 1998. **838**: p. 95-107.
49. Cochrane. *Data extraction forms. 2019*. 24/10/2019]; Available from: https://dplp.cochrane.org/data-extraction-forms.
50. NIH. *Study Quality Assessment Tools: Quality Assessment Tool for Observational Cohort and Cross-Sectional Studies*. 24/10/2019]; Available from: https://www.nhlbi.nih.gov/health-topics/study-quality-assessment-tools.
51. Iorio, A., et al., *Use of GRADE for assessment of evidence about prognosis: rating confidence in estimates of event rates in broad categories of patients.* BMJ : British Medical Journal, 2015. **350**: p. h870.

For peer review only - http://bmjopen.bmj.com/site/about/guidelines.xhtml

Appendices

Appendix 1: Search terms

Intracranial pressure, intracranial hypertension, papilloedema, optical coherence tomography

1. Intracranial pressure
2. ICP
3. Intracerebral pressure
4. Intracranial hypertension
5. Cerebral swelling
6. Cerebral oedema
7. Cerebral edema
8. Brain swelling
9. Brain oedema
10. Brain edema
11. Papilloedema
12. Papilledema
13. Optical coherence tomography
14. OCT

Search combination:

1 OR 2 OR 3 OR 4 OR 5 OR 6 OR 7 OR 8 OR 9 OR 10 OR 11 OR 12

AND

13 or 14

For peer review only

Appendix 2: Screening questions

Instructions for screeners: Tick the appropriate box per screening question. If "yes" at Stage 1, proceed to Stage 2; if "yes" at Stage 2, include. If "no" at any stage, exclude. If "unclear", proceed to next stage. If still "unclear" after Stage 2, then submit to third arbitrator for verdict.

Stage 1: Title Screening

Does the study represent Level IV evidence or above, i.e. case series, cohort studies, case-control studies, randomised controlled trials (RCTs) and systematic reviews?

Yes	
No	
Unclear	

Does the study pertain to the use of optical coherence tomography (OCT) to detect raised intracranial pressure (ICP) in children, defined as 0 to 16 years of age?

Yes	
No	
Unclear	

Stage 2: Abstract screening

Does the study represent Level IV evidence or above, i.e. case series, cohort studies, case-control studies, randomised controlled trials (RCTs) and systematic reviews?

Yes	
No	
Unclear	

Does the study pertain to the use of optical coherence tomography (OCT) to detect raised intracranial pressure (ICP) in children, defined as 0 to 16 years of age?

Yes	
No	
Unclear	

Stage 3: Full paper screening

Does the study pertain to the use of optical coherence tomography (OCT) to detect raised intracranial pressure (ICP) in children, defined as 0 to 16 years of age?

Yes	
No	
Unclear	

For peer review only

Appendix 3: Data extraction tool, adapted from the Cochrane Collaboration

General Information

Study title:
Author(s):
Source:
Date of Extraction:

Methods

Aim of study	
Study design	
Inclusion criteria	
Exclusion criteria	
Method of recruitment	
Method of randomisation (if applicable)	
Blinding (if applicable)	
Study duration	
Ethics approval	

Participants

Population description	
Setting	
Total number randomised	
Mean age	
Male/Female %	

Intervention 1

Group name	
Number in group	
Description	
Intervention parameter 1	
Intervention parameter 2 (if applicable)	
Intervention parameter 3 (if applicable)	
Intervention parameter 4 (if applicable)	
Intervention parameter 5 (if applicable)	
Intervention parameter 6 (if applicable)	
Intervention parameter 7 (if applicable)	
Intervention parameter 8 (if applicable)	
Drop-outs from group	

Outcome 1

Outcome group name	
Time points measured	

For peer review only

Outcome definition	
Outcome parameter 1	
Outcome parameter 2 (if applicable)	
Outcome parameter 3 (if applicable)	
Outcome parameter 4 (if applicable)	
Outcome parameter 5 (if applicable)	
Outcome parameter 6 (if applicable)	
Outcome parameter 7 (if applicable)	
Outcome parameter 8 (if applicable)	
Person measuring	
Imputation of missing data	

Intervention 2 (if applicable)

Intervention group name	
Number in group	
Description	
Intervention parameter 1	
Intervention parameter 2 (if applicable)	
Intervention parameter 3 (if applicable)	
Intervention parameter 4 (if applicable)	
Intervention parameter 5 (if applicable)	
Intervention parameter 6 (if applicable)	
Intervention parameter 7 (if applicable)	
Intervention parameter 8 (if applicable)	
Drop-outs from group	

For peer review only

Outcome 2 (if applicable)

Outcome group name	
Time points measured	
Outcome definition	
Outcome parameter 1	
Outcome parameter 2 (if applicable)	
Outcome parameter 3 (if applicable)	
Outcome parameter 4 (if applicable)	
Outcome parameter 5 (if applicable)	
Outcome parameter 6 (if applicable)	
Outcome parameter 7 (if applicable)	
Outcome parameter 8 (if applicable)	
Person measuring	
Imputation of missing data	

Intervention 3 (if applicable)

Intervention group name	
Number in group	

Description	
Intervention parameter 1	
Intervention parameter 2 (if applicable)	
Intervention parameter 3 (if applicable)	
Intervention parameter 4 (if applicable)	
Intervention parameter 5 (if applicable)	
Intervention parameter 6 (if applicable)	
Intervention parameter 7 (if applicable)	
Intervention parameter 8 (if applicable)	
Drop-outs from group	

Outcome 3 (if applicable)

Outcome group name	
Time points measured	
Outcome definition	
Outcome parameter 1	
Outcome parameter 2 (if applicable)	
Outcome parameter 3 (if applicable)	
Outcome parameter 4 (if applicable)	
Outcome parameter 5 (if applicable)	
Outcome parameter 6 (if applicable)	
Outcome parameter 7 (if applicable)	
Outcome parameter 8 (if applicable)	
Person measuring	
Imputation of missing data	

For peer review only

Appendix 4: NIH Quality Assessment Tool for Observational Cohort and Cross-Sectional Studies

**Criteria**	**Yes**	**No**	**Other (CD, NR, NA)***
1. Was the research question or objective in this paper clearly stated?			
2. Was the study population clearly specified and defined?			
3. Was the participation rate of eligible persons at least 50%?			
4. Were all the subjects selected or recruited from the same or similar populations (including the same time period)? Were inclusion and exclusion criteria for being in the study prespecified and applied uniformly to all participants?			
5. Was a sample size justification, power description, or variance and effect estimates provided?			
6. For the analyses in this paper, were the exposure(s) of interest measured prior to the outcome(s) being measured?			
7. Was the timeframe sufficient so that one could reasonably expect to see an association between exposure and outcome if it existed?			
8. For exposures that can vary in amount or level, did the study examine different levels of the exposure as related to the outcome (e.g., categories of exposure, or exposure measured as continuous variable)?			
9. Were the exposure measures (independent variables) clearly defined, valid, reliable, and implemented consistently across all study participants?			
10. Was the exposure(s) assessed more than once over time?			
11. Were the outcome measures (dependent variables) clearly defined, valid, reliable, and implemented consistently across all study participants?			
12. Were the outcome assessors blinded to the exposure status of participants?			
13. Was loss to follow-up after baseline 20% or less?			
14. Were key potential confounding variables measured and adjusted statistically for their impact on the relationship between exposure(s) and outcome(s)?			

*CD, cannot determine; NA, not applicable; NR, not reported

**Quality Rating (Good, Fair, or Poor)**	
**Rater #1 initials:**	
**Rater #2 initials:**	
**Additional Comments (If POOR, please state why):**	

For peer review only

PRISMA-P (Preferred Reporting Items for Systematic review and Meta-Analysis Protocols) 2015 checklist: recommended items to address in a systematic review protocol*

Section and topic	Item No	Checklist item	(Page No.#)
**ADMINISTRATIVE INFORMATION**			
Title:			
Identification	1a	Identify the report as a protocol of a systematic review	1
Update	1b	If the protocol is for an update of a previous systematic review, identify as such	1
Registration	2	If registered, provide the name of the registry (such as PROSPERO) and registration number	2
Authors:			
Contact	3a	Provide name, institutional affiliation, e-mail address of all protocol authors; provide physical mailing address of corresponding author	1
Contributions	3b	Describe contributions of protocol authors and identify the guarantor of the review	8
Amendments	4	If the protocol represents an amendment of a previously completed or published protocol, identify as such and list changes; otherwise, state plan for documenting important protocol amendments	N/A
Support:			
Sources	5a	Indicate sources of financial or other support for the review	1
Sponsor	5b	Provide name for the review funder and/or sponsor	
Role of sponsor or funder	5c	Describe roles of funder(s), sponsor(s), and/or institution(s), if any, in developing the protocol	
**INTRODUCTION**			
Rationale	6	Describe the rationale for the review in the context of what is already known	3-4
Objectives	7	Provide an explicit statement of the question(s) the review will address with reference to participants, interventions, comparators, and outcomes (PICO)	4-5
**METHODS**			
Eligibility criteria	8	Specify the study characteristics (such as PICO, study design, setting, time frame) and report characteristics (such as years considered, language, publication status) to be used as criteria for eligibility for the review	4-5
Information sources	9	Describe all intended information sources (such as electronic databases, contact with study authors, trial registers or other grey literature sources) with planned dates of coverage	6
Search strategy	10	Present draft of search strategy to be used for at least one electronic database, including planned limits, such that it could be repeated	6 and Appendix-1

For peer review only

Study records:			
Data management	11a	Describe the mechanism(s) that will be used to manage records and data throughout the review	6-7
Selection process	11b	State the process that will be used for selecting studies (such as two independent reviewers) through each phase of the review (that is, screening, eligibility and inclusion in meta-analysis)	6 and Appendix 2
Data collection process	11c	Describe planned method of extracting data from reports (such as piloting forms, done independently, in duplicate), any processes for obtaining and confirming data from investigators	6-7 and Appendix 3
Data items	12	List and define all variables for which data will be sought (such as PICO items, funding sources), any pre-planned data assumptions and simplifications	6-7
Outcomes and prioritization	13	List and define all outcomes for which data will be sought, including prioritization of main and additional outcomes, with rationale	6-7
Risk of bias in individual studies	14	Describe anticipated methods for assessing risk of bias of individual studies, including whether this will be done at the outcome or study level, or both; state how this information will be used in data synthesis	7 and Appendix 4
Data synthesis	15a	Describe criteria under which study data will be quantitatively synthesised	6-7
	15b	If data are appropriate for quantitative synthesis, describe planned summary measures, methods of handling data and methods of combining data from studies, including any planned exploration of consistency (such as I^2, Kendall's τ)	N/A
	15c	Describe any proposed additional analyses (such as sensitivity or subgroup analyses, meta-regression)	7
	15d	If quantitative synthesis is not appropriate, describe the type of summary planned	7
Meta-bias(es)	16	Specify any planned assessment of meta-bias(es) (such as publication bias across studies, selective reporting within studies)	7
Confidence in cumulative evidence	17	Describe how the strength of the body of evidence will be assessed (such as GRADE)	7

*** It is strongly recommended that this checklist be read in conjunction with the PRISMA-P Explanation and Elaboration (cite when available) for important clarification on the items. Amendments to a review protocol should be tracked and dated. The copyright for PRISMA-P (including checklist) is held by the PRISMA-P Group and is distributed under a Creative Commons Attribution Licence 4.0.**

From: Shamseer L, Moher D, Clarke M, Ghersi D, Liberati A, Petticrew M, Shekelle P, Stewart L, PRISMA-P Group. Preferred reporting items for systematic review and meta-analysis protocols (PRISMA-P) 2015: elaboration and explanation. BMJ. 2015 Jan 2;349(jan02 1):g7647.

For peer review only

BMJ Open

Detection of Intracranial Hypertension in Children using Optical Coherence Tomography: A Systematic Review Protocol

Journal:	*BMJ Open*
Manuscript ID	bmjopen-2020-037833.R1
Article Type:	Protocol
Date Submitted by the Author:	17-Apr-2020

Complete List of Authors:	Rufai, Sohaib; Great Ormond Street Hospital For Children NHS Foundation Trust, Clinical and Academic Department of Ophthalmology; University of Leicester, University of Leicester Ulverscroft Eye Unit<br>Jeelani, Noor ul Owase; Great Ormond Street Hospital For Children NHS Foundation Trust, Department of Neurosurgery<br>McLean, Rebecca; University of Leicester, University of Leicester Ulverscroft Eye Unit

**Primary Subject Heading**:	Ophthalmology
Secondary Subject Heading:	Evidence based practice, Neurology, Paediatrics, Surgery
Keywords:	OPHTHALMOLOGY, Neuro-ophthalmology < OPHTHALMOLOGY, Paediatric ophthalmology < OPHTHALMOLOGY, PAEDIATRICS, NEUROSURGERY

SCHOLARONE™ Manuscripts

BMJ

I, the Submitting Author has the right to grant and does grant on behalf of all authors of the Work (as defined in the below author licence), an exclusive licence and/or a non-exclusive licence for contributions from authors who are: i) UK Crown employees; ii) where BMJ has agreed a CC-BY licence shall apply, and/or iii) in accordance with the terms applicable for US Federal Government officers or employees acting as part of their official duties; on a worldwide, perpetual, irrevocable, royalty-free basis to BMJ Publishing Group Ltd ("BMJ") its licensees and where the relevant Journal is co-owned by BMJ to the co-owners of the Journal, to publish the Work in this journal and any other BMJ products and to exploit all rights, as set out in our licence.

The Submitting Author accepts and understands that any supply made under these terms is made by BMJ to the Submitting Author unless you are acting as an employee on behalf of your employer or a postgraduate student of an affiliated institution which is paying any applicable article publishing charge ("APC") for Open Access articles. Where the Submitting Author wishes to make the Work available on an Open Access basis (and intends to pay the relevant APC), the terms of reuse of such Open Access shall be governed by a Creative Commons licence – details of these licences and which Creative Commons licence will apply to this Work are set out in our licence referred to above.

Other than as permitted in any relevant BMJ Author's Self Archiving Policies, I confirm this Work has not been accepted for publication elsewhere, is not being considered for publication elsewhere and does not duplicate material already published. I confirm all authors consent to publication of this Work and authorise the granting of this licence.

For peer review only

Detection of Intracranial Hypertension in Children using Optical Coherence Tomography: A Systematic Review Protocol

Authors:

Dr Sohaib R. Rufai[1,2] (BMBS, MRes), Mr Noor ul Owase Jeelani[3,4] (MPhil, FRCS), Dr Rebecca J. McLean[1] (MSc, PhD)

Affiliations:

1. The University of Leicester Ulverscroft Eye Unit, Robert Kilpatrick Clinical Sciences Building, Leicester Royal Infirmary, Leicester, UK.
2. Clinical and Academic Department of Ophthalmology, Great Ormond Street Hospital for Children NHS Trust, London, UK.
3. Department of Neurosurgery, Great Ormond Street Hospital for Children NHS Trust, London, UK.
4. Developmental Biology & Cancer Department, UCL GOS Institute of Child Health, Faculty of Population Health Sciences, UCL, London, UK

Corresponding Author*

Dr Rebecca J. McLean (MSc, PhD)

The University of Leicester Ulverscroft Eye Unit, Robert Kilpatrick Clinical Sciences Building, PO Box 65, Leicester LE2 7LX, UK

E-mail: rjm19@leicester.ac.uk

Telephone: +44 (0)116 2523268

Fax: +44 (0)116 2231996

Funding: The Medical Research Council (London, UK) (grant no.: MR/N004566/1). SRR is funded by a National Institute for Health Research (NIHR) Doctoral Fellowship for this research project. This paper presents independent research funded by the National Institute for Health Research (NIHR) and MRC. The views expressed are those of the author(s) and not necessarily those of the MRC, the NHS, the NIHR or the Department of Health and Social Care.

Sponsor: University of Leicester

Role of sponsor or funder: The sponsor and funder had no role in developing this systematic review protocol.

Competing interests: The authors have no proprietary or commercial interest in any materials discussed in this study.

Keywords: Intracranial pressure, intracranial hypertension, optical coherence tomography, early recognition, screening, systematic review protocol

Word Count: 1760

For peer review only

For peer review only - http://bmjopen.bmj.com/site/about/guidelines.xhtml

Abstract

Introduction

Intracranial hypertension in children can have deleterious effects on the brain and vision. It is notoriously difficult to estimate intracranial pressure (ICP) in children and existing methods deliver sub-optimal diagnostic accuracy to be used as screening tools. Optical coherence tomography (OCT) may represent a valuable, non-invasive surrogate measure of ICP, as has been demonstrated in a number of associated conditions affecting adults. More recently, OCT has been employed within the paediatric age group. However, the role of OCT in detecting intracranial hypertension (ICH) in children has not been rigorously assessed in a systematic review for all relevant conditions. Here, we propose a systematic review protocol to examine the role of OCT in the detection of ICH in children.

Methods and analysis

Electronic searches in the Cochrane Central Register of Controlled Trials (CENTRAL), MEDLINE, Embase, Web of Science and PubMed will identify studies featuring OCT in detecting ICH in children. Two independent screeners will identify studies for inclusion using a screening questionnaire. The systematic search and screening will take place between 2nd April 2020 and 1st June 2020, whilst we aim to complete data analysis by 1st September 2020. Quality assessment will be performed using the National Institutes of Health (NIH) Quality Assessment Tool for Observational Cohort and Cross-Sectional Studies. The primary outcome measure is the sensitivity and specificity of OCT in detecting ICH in children. Secondary outcomes measures include conditions associated with ICH per study, direct ICP monitoring, sensitivity and specificity of other measures for ICP and OCT parameters used.

Ethics and dissemination: Ethical approval is not required for the proposed systematic review as no primary data will be collected. The findings will be disseminated through presentations at scientific meetings and peer-reviewed journal publication.

Trial registration number: International Prospective Register for Systematic Reviews (PROSPERO) number CRD42019154254

Strengths and limitations of this study

- The proposed study will be the first to examine the role of optical coherence tomography for all paediatric conditions associated with intracranial hypertension.
- This protocol provides transparency to the proposed systematic review methodology and reduces the possibility of duplication.
- The proposed methodology is recommended by the Cochrane Collaboration.
- A scarcity of relevant randomised controlled trials and associated high risk of bias of observational studies may be the main limitations in the evidence returned by this systematic review.
- The broad search strategy may result in a large number of titles and abstracts to screen initially.

For peer review only - http://bmjopen.bmj.com/site/about/guidelines.xhtml

Introduction

Intracranial hypertension

Intracranial hypertension (ICH) was first described by Quincke in 1896 and remains a subject of major importance among the medical profession.[1] ICH can pose devastating consequences if untreated, including visual impairment, neurocognitive delay, disability and death.[2, 3] Subacute conditions in children can cause insidious elevation of intracranial pressure (ICP) which may go undetected before insult to the brain and vision has already begun.[2, 4] Hence, early detection of ICH is crucial in enabling timely medical intervention to prevent or limit the sequelae of ICH.

ICH affects between 0.63 and 0.71 per 100,000 children.[5, 6] ICH can be broadly classified as primary (or idiopathic) and secondary. Primary ICH is characterised by elevated ICP with no identifiable cause.[4, 7] Common risk factors for primary ICH include female gender, obesity and postpubertal status, although it is known to occur in children with a lack of predilection for gender and obesity.[4, 8-14] Secondary ICH describes elevated ICP that is a direct result of another condition, for example an expansive intracranial process, such as space occupying lesion or hydrocephalus, or a constricting skull pathology, such as the premature fusion of cranial sutures in craniosynostosis.[2-4]

Measurement of intracranial pressure

ICP measurement in children is notoriously difficult. The gold standard measure is direct intraparenchymal ICP measurement, which involves overnight admission to hospital and general anaesthesia. The risks of intraparenchymal ICP measurement include infection, haemorrhage, CSF leak and device failure.[15, 16] Furthermore, there is no universal agreement on timing, duration and frequency for accurate measurement or indeed agreement on the normal paediatric range of ICP – 11-15mmHg is widely considered normal, but Hayward *et al* argue that the upper limit could reasonably be raised to 20mmHg.[17, 18]

The ideal screening method for ICH should be sensitive, specific and child-friendly with the ability to record objective serial measurements over time. However, existing methods fail to satisfy all of these criteria. Visual evoked potentials (VEP) assess the amplitude and latency time of the averaged encephalographic response to visual stimulus. Axonal injury secondary to ICH is associated with reduction of amplitude or prolongation of the latency time.[2, 19] However, limitations of VEP include good the need for cooperation by the child, plus high variability in normal subjects.[20] Fundus examination can permit direct visualisation of papilloedema or optic atrophy, both of which are associated with ICH. A study by Tuite *et al* demonstrated that fundus examination had sensitivity of 100% in detecting ICH in children aged over 8 years, but only 22% for that in younger patients, excluding it as a suitable screening method. B-scan ocular ultrasound can be helpful in acute situations for detecting severe ICH, but possesses a sensitivity of only 11% when compared to fundoscopy.[21-24] Radiological signs, such as the “beaten copper” appearance of the cranium, universally demonstrate poor sensitivity for ICH.[25]

Optical coherence tomography

The optic nerve is primarily intracranial. Thus, ICH causes optic nerve head swelling, termed papilloedema, with secondary retinal changes. These changes can be measured objectively using optical coherence tomography (OCT) – a non-invasive imaging technique that can acquire ultra-high resolution, three-dimensional images of the back of the eye within seconds.[26] OCT has been used to describe the normal development of the optic nerve[27] and fovea[27, 28] in children. OCT has also been

For peer review only - http://bmjopen.bmj.com/site/about/guidelines.xhtml

utilised in a wide range of conditions within paediatric ophthalmology, including retinopathy of prematurity,[29] retinoblastoma,[30] nystagmus[31], albinism[32], achromatopsia[33], foveal hypoplasia,[34] optic nerve hypoplasia,[35] primary congenital glaucoma[36], microcephaly[37] and others. Furthermore, OCT has been successfully utilised as a diagnostic tool in a variety of conditions associated with ICH, including idiopathic intracranial hypertension, craniosynostosis and hydrocephalus.[38-44]

Thus, OCT may represent an ideal screening method for ICH in children owing to its quick acquisition time and non-invasive, child-friendly nature. However, this has not yet been rigorously assessed in a systematic review. Here, we propose to conduct the first systematic review examining the role of OCT in all conditions associated with ICH in children.

Objectives

The primary research objective of this proposed systematic review is to assess the sensitivity and specificity of OCT in detecting ICH in children.

The secondary research objectives are as follows:

- To assess which conditions associated with ICH have been successfully studied by OCT;
- To assess OCT success rate per condition per study;
- To identify surrogate measures of ICP and their associated sensitivity, specificity and success rates;
- To assess which ICP range is determined as normal per condition per study.

Methods

This protocol has adhered to the Preferred Reporting Items for Systematic review and Meta-Analysis Protocols (PRISMA-P) checklist.[45]

Eligibility criteria

The Population, Intervention, Comparison, Outcome and Study design (PICOS) strategy[46] has been employed to outline the eligibility criteria for this systematic review, as summarised in Table 1.

PICOS strategy[46]	Inclusion criteria	Exclusion criteria
P - Population	Studies of children, defined as being aged under 18 years, diagnosed with conditions associated with ICH.	i) Studies of adults aged 18 years old or over;<br>ii) Studies not pertaining to the diagnosis of ICH.
I - Intervention	Studies employing OCT to detect ICH.	Any studies that do not include OCT.
C - Comparator	Absence of a comparator will not lead to exclusion of studies, as it may be unethical to deprive one arm of the study of OCT when it may lead to a better clinical outcome in such a dangerous situation of ICH.	N/A

For peer review only - http://bmjopen.bmj.com/site/about/guidelines.xhtml

O - Outcome	Sensitivity and specificity of any OCT measure(s), ± surrogate measure(s), for ICH.	Studies that do not report OCT measures.
S - Study design	All Level IV evidence and above, i.e. systematic reviews, randomised controlled trials (RCTs), cohort studies, case-control studies and case series, as defined by the Oxford Centre for Evidence-based Medicine.[47]	Level 5 evidence, i.e. expert opinion without critical appraisal.

Table 1: Eligibility criteria. Key: ICP = intracranial pressure; OCT = optical coherence tomography; PICOS = Population, Intervention, Comparison, Outcome and Study design.

Types of outcome measures

The main outcome measure will be sensitivity and specificity for OCT measures in detecting ICH. OCT metrics may include one or more of the following:

- Optic nerve parameters: cup depth, cup width, cup volume, disc width and cup to disc ratio;
- Rim parameters: retinal nerve fibre layer (RNFL) thickness, rim area, rim volume, Bruch's membrane opening-minimum rim width (BMO-MRW), Bruch's membrane orientation, ganglion cell layer thickness, full peripapillary analysis;
- Retinal parameters: macular and perimacular retinal thickness, foveal pit width, depth and area, plus segmentation of all retinal layers.

The secondary outcome measures are as follows:

- Condition(s) associated with ICH per study;
- OCT success rate;
- Other surrogate estimates of ICP;
- Other OCT parameters not listed above;
- ICP range determined as normal.

Quality of life outcomes

Quality of life outcomes and patient satisfaction measured by surveys will be included where possible.

Adverse events

The process of OCT imaging cannot directly produce adverse events as it is a safe and non-invasive imaging method that uses low-coherence light.[48] It does not involve ionising radiation.

Follow-up

There will be no minimum follow-up period set for study inclusion. In many cases, the decision to employ rapid intervention (i.e. surgery) is made as soon as possible once ICH has been diagnosed. The stipulation of a minimum follow-up period would therefore risk losing valuable data from excluded studies.

For peer review only - http://bmjopen.bmj.com/site/about/guidelines.xhtml

Information sources

Record characteristics

There shall be no time restriction on records considered for this study. Unpublished records shall be considered. There shall be no language restrictions; all applicable non-English records will undergo professional translation from the University of Leicester Centre for Translation and Interpreting Studies.

Electronic searches

The following platforms shall be searched:

- Cochrane Central Register of Controlled Trials (CENTRAL) (including the Cochrane Eyes and Vision Group (CEVG) Trials Register)
- EMBASE Classic+Embase (1947 to present)
- Ovid MEDLINE(R) (1946 to present)
- Ovid MEDLINE In-Process and Other Non-Indexed Citations
- PubMed (1948 to present)
- Web of Science Core Collection (1970 to present)

Searching other resources

Unpublished studies identified by experts will be considered against our inclusion criteria. References of included studies will be searched and the authors contacted by e-mail or letter to identify further unpublished works where applicable. If the authors do not reply within 14 days, a reminder will be sent by e-mail or letter. If the authors do not reply to the reminder within 14 days, their study will be excluded.

Search strategy

Medical subject headings (MeSH) terms for 'intracranial hypertension' and 'optical coherence tomography' where entered into the above search platforms. Full details of search terms and strategy are included in Appendix 1.

Study records

Data management

EndNote X9 (Thomson Reuters, New York, NY) reference management software will be used for data management.

Selection of studies

Two independent screeners (SRR and RJM) shall follow a three-stage screening method. The search will be conducted on 2nd April 2020 and screening will be performed until 1st June 2020. Appendix 2 outlines the screening questions and process. First, titles will be screened, followed by abstracts, followed by full papers. At each stage, studies shall be classified as 'include', 'exclude' or 'unclear', with those not excluded progressing to the next stage of screening. If any studies are classified as 'unclear' following the full paper screen, the study authors in question would be contacted by e-mail or letter for clarification. If the authors fail to reply within 14 days, a reminder will be sent. If the authors fail to reply to the reminder within 14 days, their study will be excluded. If there is any

For peer review only - http://bmjopen.bmj.com/site/about/guidelines.xhtml

disagreement over which papers are selected for inclusion, the third arbitrator (NUOJ) shall make the final decision.

Data collection process

Our data extraction tool is included in Appendix 3, which is adapted from the Cochrane Collaboration.[49]

Data items, outcomes and prioritisation

Table 2 outlines the data items to be extracted. Extraction of primary outcome measures will be addressed as these will be used to address the primary research objective of this systematic review.

Data category	Data to be extracted
Study characteristics	• Author(s)<br>• Year<br>• Study design<br>• Study location<br>• Number of patients<br>• Mean age<br>• Age range
Primary outcome measures	• OCT Parameter(s)<br>○ Sensitivity (%)<br>○ Specificity (%)
Secondary outcome measures, where available	• Condition(s) studies;<br>• OCT success rate;<br>• Other surrogate estimates of ICP;<br>○ Sensitivity (%)<br>○ Specificity (%)<br>• Other OCT parameters not listed above;<br>• ICP range determined as normal.<br>• Quality of life outcomes<br>• Adverse events

Table 2: Data to be extracted. Key: ICP = intracranial pressure; OCT = optical coherence tomography.

For peer review only

Risk of bias in individual studies

The National Institutes of Health (NIH) Quality Assessment Tool for Observational Cohort and Cross-Sectional Studies,[50] included in Appendix 4, shall be used to assess risk of bias in the included studies.

Data analysis

Scoping searches suggest that mainly observational studies will be returned by our search strategy with few relevant RCTs. Weighted means for outcome measures will only be calculated if multiple RCTs are identified. Otherwise, we shall perform a qualitative review summarising the available evidence. A narrative synthesis will be conducted, examining the strengths, weaknesses, similarities and differences between included studies. Outcomes measures will be extracted and reported

For peer review only - http://bmjopen.bmj.com/site/about/guidelines.xhtml

descriptively. Included studies will be evaluated based on the strength of the evidence. We aim to complete data analysis by 1st September 2020.

Confidence in cumulative evidence

The Grades of Recommendation, Assessment, Development, and Evaluation (GRADE) approach[51] will be employed to evaluate the strength of the body of evidence identified in this study, where applicable.

Patient and public involvement

Involvement of patients and the public in the development of this systematic protocol is not applicable, as no patient recruitment will take place.

Ethics and Dissemination

Ethical approval is not required for the proposed systematic review as no primary data will be collected. The findings of this systematic review shall be disseminated through presentations at scientific meetings, as well as peer-reviewed, open access journal publication. Data generated during this systematic review will be made available from the corresponding author on reasonable request.

Discussion

To the best of our knowledge, this is the first proposed systematic review to rigorously examine the role of OCT in detecting ICH in children. We have adhered to the PRISMA-P checklist[45] when developing this protocol. Two independent screeners shall conduct the search, with a third arbitrator available if required. Risk of bias will be assessed using the NIH Quality Assessment Tool for Observational Cohort and Cross-Sectional Studies.[50] This systematic review should help to clarify the role of OCT as a non-invasive screening method for ICH in paediatrics. The main limitations of the study may be due to the low number of relevant RCTs and higher risk of bias associated with observational studies returned by the systematic review. Publication of this protocol should provide transparency to our proposed systematic review methodology as well as reduce the possibility of duplication.

Acknowledgements

We would like to thank Prof. Irene Gottlob and Dr. Frank Proudlock of the University of Leicester Ulverscroft Eye Unit, for their support and contributions towards this systematic review protocol.

Author Contributions

SRR: Concept, methodology, protocol writing, final approval.

NUOJ: Supervision, concept, critical revision, final approval.

RJM: Supervision, methodology, critical revision, final approval, guarantor of review.

For peer review only - http://bmjopen.bmj.com/site/about/guidelines.xhtml

References

1. Quincke, H., *Ueber Meningitis serosa und verwandte Zustände.* Deutsche Zeitschrift für Nervenheilkunde, 1896. **9**(3-4): p. 149-168.
2. Derderian, C. and J. Seaward, *Syndromic craniosynostosis.* Seminars in plastic surgery, 2012. **26**(2): p. 64-75.
3. Jordan, C.O. and S.C. Aylward, *Intracranial hypertension: a current review.* Curr Opin Pediatr, 2018. **30**(6): p. 764-774.
4. Aylward, S.C. and R.E. Reem, *Pediatric Intracranial Hypertension.* Pediatric Neurology, 2017. **66**: p. 32-43.
5. Matthews, Y.Y., et al., *Pseudotumor cerebri syndrome in childhood: incidence, clinical profile and risk factors in a national prospective population-based cohort study.* Arch Dis Child, 2017. **102**(8): p. 715-721.
6. Gillson, N., et al., *Incidence and Demographics of Pediatric Intracranial Hypertension.* Pediatr Neurol, 2017. **73**: p. 42-47.
7. Mollan, S.P., et al., *Idiopathic intracranial hypertension: consensus guidelines on management.* J Neurol Neurosurg Psychiatry, 2018. **89**(10): p. 1088-1100.
8. Radhakrishnan, K., et al., *Epidemiology of idiopathic intracranial hypertension: a prospective and case-control study.* J Neurol Sci, 1993. **116**(1): p. 18-28.
9. Raoof, N., et al., *The incidence and prevalence of idiopathic intracranial hypertension in Sheffield, UK.* Eur J Neurol, 2011. **18**(10): p. 1266-8.
10. Radhakrishnan, K., et al., *Idiopathic intracranial hypertension (pseudotumor cerebri). Descriptive epidemiology in Rochester, Minn, 1976 to 1990.* Arch Neurol, 1993. **50**(1): p. 78-80.
11. Cinciripini, G.S., S. Donahue, and M.S. Borchert, *Idiopathic intracranial hypertension in prepubertal pediatric patients: characteristics, treatment, and outcome.* Am J Ophthalmol, 1999. **127**(2): p. 178-82.
12. Kesler, A. and A. Fattal-Valevski, *Idiopathic intracranial hypertension in the pediatric population.* J Child Neurol, 2002. **17**(10): p. 745-8.
13. Babikian, P., J. Corbett, and W. Bell, *Idiopathic intracranial hypertension in children: the Iowa experience.* J Child Neurol, 1994. **9**(2): p. 144-9.
14. Phillips, P.H., M.X. Repka, and S.R. Lambert, *Pseudotumor cerebri in children.* J aapos, 1998. **2**(1): p. 33-8.
15. Zhang, X., et al., *Invasive and noninvasive means of measuring intracranial pressure: a review.* Physiol Meas, 2017. **38**(8): p. R143-r182.
16. Tamburrini, G., et al., *Intracranial pressure monitoring in children with single suture and complex craniosynostosis: a review.* Childs Nerv Syst, 2005. **21**(10): p. 913-21.
17. Renier, D., et al., *Intracranial pressure in craniostenosis.* J Neurosurg, 1982. **57**(3): p. 370-7.
18. Hayward, R., et al., *Connecting raised intracranial pressure and cognitive delay in craniosynostosis: many assumptions, little evidence.* J Neurosurg Pediatr, 2016. **18**(2): p. 242-50.
19. Thompson, D.A., et al., *Prevalence of abnormal pattern reversal visual evoked potentials in craniosynostosis.* Plast Reconstr Surg, 2006. **118**(1): p. 184-92.
20. Andersson, L., J. Sjölund, and J. Nilsson, *Flash visual evoked potentials are unreliable as markers of ICP due to high variability in normal subjects.* Acta Neurochirurgica, 2012. **154**(1): p. 121-127.
21. Dubourg, J., et al., *Ultrasonography of optic nerve sheath diameter for detection of raised intracranial pressure: a systematic review and meta-analysis.* Intensive Care Med, 2011. **37**(7): p. 1059-68.

For peer review only - http://bmjopen.bmj.com/site/about/guidelines.xhtml

22. Driessen, C., et al., *Nocturnal ultrasound measurements of optic nerve sheath diameter correlate with intracranial pressure in children with craniosynostosis.* Plast Reconstr Surg, 2012. **130**(3): p. 448e-51e.
23. Bolesch, S., et al., *Transcranial ultrasound to detect elevated intracranial pressure: comparison of septum pellucidum undulations and optic nerve sheath diameter.* Ultrasound Med Biol, 2015. **41**(5): p. 1233-40.
24. Driessen, C., et al., *Are ultrasonography measurements of optic nerve sheath diameter an alternative to funduscopy in children with syndromic craniosynostosis?* J Neurosurg Pediatr, 2011. **8**(3): p. 329-34.
25. Tuite, G.F., et al., *The beaten copper cranium: a correlation between intracranial pressure, cranial radiographs, and computed tomographic scans in children with craniosynostosis.* Neurosurgery, 1996. **39**(4): p. 691-9.
26. Lee, H., F.A. Proudlock, and I. Gottlob, *Pediatric Optical Coherence Tomography in Clinical Practice-Recent Progress.* Invest Ophthalmol Vis Sci, 2016. **57**(9): p. OCT69-79.
27. Patel, A., et al., *Optic Nerve Head Development in Healthy Infants and Children Using Handheld Spectral-Domain Optical Coherence Tomography.* Ophthalmology, 2016. **123**(10): p. 2147-57.
28. Lee, H., et al., *In Vivo Foveal Development Using Optical Coherence Tomography.* Invest Ophthalmol Vis Sci, 2015. **56**(8): p. 4537-45.
29. Anwar, S., et al., *POTENTIAL UTILITY OF FOVEAL MORPHOLOGY IN PRETERM INFANTS MEASURED USING HAND-HELD OPTICAL COHERENCE TOMOGRAPHY IN RETINOPATHY OF PREMATURITY SCREENING.* Retina (Philadelphia, Pa.), 2019: p. 10.1097/IAE.0000000000002622.
30. Soliman, S.E., et al., *Optical Coherence Tomography-Guided Decisions in Retinoblastoma Management.* Ophthalmology, 2017. **124**(6): p. 859-872.
31. Lee, H., F. Proudlock, and I. Gottlob, *Is handheld optical coherence tomography reliable in infants and young children with and without nystagmus?* Investigative ophthalmology & visual science, 2013. **54**(13): p. 8152-8159.
32. Mohammad, S., et al., *Characterization of Abnormal Optic Nerve Head Morphology in Albinism Using Optical Coherence Tomography.* Investigative ophthalmology & visual science, 2015. **56**(8): p. 4611-4618.
33. Thomas, M.G., et al., *High-resolution in vivo imaging in achromatopsia.* Ophthalmology, 2011. **118**(5): p. 882-887.
34. Rufai, S.R., et al., *Can Structural Grading of Foveal Hypoplasia Predict Future Vision in Infantile Nystagmus?: A Longitudinal Study.* Ophthalmology, 2019: p. S0161-6420(19)32212-2.
35. Pilat, A., et al., *High-Resolution Imaging of the Optic Nerve and Retina in Optic Nerve Hypoplasia.* Ophthalmology, 2015. **122**(7): p. 1330-1339.
36. Pilat, A.V., et al., *Assessment of the anterior segment of patients with primary congenital glaucoma using handheld optical coherence tomography.* Eye (London, England), 2019. **33**(8): p. 1232-1239.
37. Papageorgiou, E., et al., *Retinal and optic nerve changes in microcephaly: An optical coherence tomography study.* Neurology, 2018. **91**(6): p. e571-e585.
38. Optical Coherence Tomography Substudy, C. and N.I.I.H.S. Group, *Papilledema Outcomes from the Optical Coherence Tomography Substudy of the Idiopathic Intracranial Hypertension Treatment Trial.* Ophthalmology, 2015. **122**(9): p. 1939-45.e2.
39. Scott, C.J., et al., *Diagnosis and grading of papilledema in patients with raised intracranial pressure using optical coherence tomography vs clinical expert assessment using a clinical staging scale.* Archives of ophthalmology (Chicago, Ill. : 1960), 2010. **128**(6): p. 705-711.
40. Skau, M., et al., *Diagnostic value of optical coherence tomography for intracranial pressure in idiopathic intracranial hypertension.* Graefe's archive for clinical and experimental

For peer review only - http://bmjopen.bmj.com/site/about/guidelines.xhtml

ophthalmology = Albrecht von Graefes Archiv fur klinische und experimentelle Ophthalmologie, 2013. **251**(2): p. 567-574.
41. Anand, A., et al., *Optical coherence tomography of the optic nerve head detects acute changes in intracranial pressure.* Journal of clinical neuroscience : official journal of the Neurosurgical Society of Australasia, 2016. **29**: p. 73-76.
42. Patel, M.D., et al., *Methods for Quantifying Optic Disc Volume and Peripapillary Deflection Volume Using Radial Optical Coherence Tomography Scans and Association With Intracranial Pressure.* Frontiers in neurology, 2019. **10**: p. 798-798.
43. Swanson, J.W., et al., *Evaluation of Optical Coherence Tomography to Detect Elevated Intracranial Pressure in Children.* JAMA Ophthalmol, 2017. **135**(4): p. 320-328.
44. Swanson, J.W., et al., *Intracranial pressure patterns in children with craniosynostosis utilizing optical coherence tomography.* Child's nervous system : ChNS : official journal of the International Society for Pediatric Neurosurgery, 2019: p. 10.1007/s00381-019-04448-x.
45. Shamseer, L., et al., *Preferred reporting items for systematic review and meta-analysis protocols (PRISMA-P) 2015: elaboration and explanation.* BMJ : British Medical Journal, 2015. **349**: p. g7647.
46. Higgins, J.P. and S. Green, *Defining the review question and developing criteria for including studies*, in *Cochrane handbook for systematic reviews of interventions*. 2008, Wiley Online Library. p. 83.
47. CEBM. *Oxford Centre for Evidence-based Medicine – Levels of Evidence (March 2009)*. 24/10/2019]; Available from: https://www.cebm.net/2009/06/oxford-centre-evidence-based-medicine-levels-evidence-march-2009/.
48. Fujimoto, J.G., et al., *New technology for high-speed and high-resolution optical coherence tomography.* Ann N Y Acad Sci, 1998. **838**: p. 95-107.
49. Cochrane. *Data extraction forms. 2019*. 24/10/2019]; Available from: https://dplp.cochrane.org/data-extraction-forms.
50. NIH. *Study Quality Assessment Tools: Quality Assessment Tool for Observational Cohort and Cross-Sectional Studies*. 24/10/2019]; Available from: https://www.nhlbi.nih.gov/health-topics/study-quality-assessment-tools.
51. Iorio, A., et al., *Use of GRADE for assessment of evidence about prognosis: rating confidence in estimates of event rates in broad categories of patients.* BMJ : British Medical Journal, 2015. **350**: p. h870.

For peer review only - http://bmjopen.bmj.com/site/about/guidelines.xhtml

Appendices

Appendix 1: Search terms

Intracranial pressure, intracranial hypertension, papilloedema, optical coherence tomography

1. Intracranial pressure
2. ICP
3. Intracerebral pressure
4. Intracranial hypertension
5. Cerebral swelling
6. Cerebral oedema
7. Cerebral edema
8. Brain swelling
9. Brain oedema
10. Brain edema
11. Papilloedema
12. Papilledema
13. Optical coherence tomography
14. OCT

Search combination:

1 OR 2 OR 3 OR 4 OR 5 OR 6 OR 7 OR 8 OR 9 OR 10 OR 11 OR 12

AND

13 or 14

For peer review only

Appendix 2: Screening questions

Instructions for screeners: Tick the appropriate box per screening question. If "yes" at Stage 1, proceed to Stage 2; if "yes" at Stage 2, include. If "no" at any stage, exclude. If "unclear", proceed to next stage. If still "unclear" after Stage 2, then submit to third arbitrator for verdict.

Stage 1: Title Screening

Does the study represent Level IV evidence or above, i.e. case series, cohort studies, case-control studies, randomised controlled trials (RCTs) and systematic reviews?

Yes	
No	
Unclear	

Does the study pertain to the use of optical coherence tomography (OCT) to detect raised intracranial pressure (ICP) in children, defined as under 18 years of age?

Yes	
No	
Unclear	

Stage 2: Abstract screening

Does the study represent Level IV evidence or above, i.e. case series, cohort studies, case-control studies, randomised controlled trials (RCTs) and systematic reviews?

Yes	
No	
Unclear	

Does the study pertain to the use of optical coherence tomography (OCT) to detect raised intracranial pressure (ICP) in children, defined as under 18 years of age?

Yes	
No	
Unclear	

Stage 3: Full paper screening

Does the study represent Level IV evidence or above and pertain to the use of optical coherence tomography (OCT) to detect raised intracranial pressure (ICP) in children, defined as under 18 years of age?

Yes	
No	
Unclear	

For peer review only

Appendix 3: Data extraction tool, adapted from the Cochrane Collaboration

General Information

Study title:
Author(s):
Source:
Date of Extraction:

Methods

Aim of study	
Study design	
Inclusion criteria	
Exclusion criteria	
Method of recruitment	
Method of randomisation (if applicable)	
Blinding (if applicable)	
Study duration	
Ethics approval	

Participants

Population description	
Setting	
Total number randomised	
Mean age	
Male/Female %	

Intervention 1

Group name	
Number in group	
Description	
Intervention parameter 1	
Intervention parameter 2 (if applicable)	
Intervention parameter 3 (if applicable)	
Intervention parameter 4 (if applicable)	
Intervention parameter 5 (if applicable)	
Intervention parameter 6 (if applicable)	
Intervention parameter 7 (if applicable)	
Intervention parameter 8 (if applicable)	
Drop-outs from group	

Outcome 1

Outcome group name	
Time points measured	

For peer review only

Outcome definition	
Outcome parameter 1	
Outcome parameter 2 (if applicable)	
Outcome parameter 3 (if applicable)	
Outcome parameter 4 (if applicable)	
Outcome parameter 5 (if applicable)	
Outcome parameter 6 (if applicable)	
Outcome parameter 7 (if applicable)	
Outcome parameter 8 (if applicable)	
Person measuring	
Imputation of missing data	

Intervention 2 (if applicable)

Intervention group name	
Number in group	
Description	
Intervention parameter 1	
Intervention parameter 2 (if applicable)	
Intervention parameter 3 (if applicable)	
Intervention parameter 4 (if applicable)	
Intervention parameter 5 (if applicable)	
Intervention parameter 6 (if applicable)	
Intervention parameter 7 (if applicable)	
Intervention parameter 8 (if applicable)	
Drop-outs from group	

Outcome 2 (if applicable)

Outcome group name	
Time points measured	
Outcome definition	
Outcome parameter 1	
Outcome parameter 2 (if applicable)	
Outcome parameter 3 (if applicable)	
Outcome parameter 4 (if applicable)	
Outcome parameter 5 (if applicable)	
Outcome parameter 6 (if applicable)	
Outcome parameter 7 (if applicable)	
Outcome parameter 8 (if applicable)	
Person measuring	
Imputation of missing data	

Intervention 3 (if applicable)

Intervention group name	
Number in group	

For peer review only

Description	
Intervention parameter 1	
Intervention parameter 2 (if applicable)	
Intervention parameter 3 (if applicable)	
Intervention parameter 4 (if applicable)	
Intervention parameter 5 (if applicable)	
Intervention parameter 6 (if applicable)	
Intervention parameter 7 (if applicable)	
Intervention parameter 8 (if applicable)	
Drop-outs from group	

Outcome 3 (if applicable)

Outcome group name	
Time points measured	
Outcome definition	
Outcome parameter 1	
Outcome parameter 2 (if applicable)	
Outcome parameter 3 (if applicable)	
Outcome parameter 4 (if applicable)	
Outcome parameter 5 (if applicable)	
Outcome parameter 6 (if applicable)	
Outcome parameter 7 (if applicable)	
Outcome parameter 8 (if applicable)	
Person measuring	
Imputation of missing data	

For peer review only

Appendix 4: NIH Quality Assessment Tool for Observational Cohort and Cross-Sectional Studies

Criteria	Yes	No	Other (CD, NR, NA)*
1. Was the research question or objective in this paper clearly stated?			
2. Was the study population clearly specified and defined?			
3. Was the participation rate of eligible persons at least 50%?			
4. Were all the subjects selected or recruited from the same or similar populations (including the same time period)? Were inclusion and exclusion criteria for being in the study prespecified and applied uniformly to all participants?			
5. Was a sample size justification, power description, or variance and effect estimates provided?			
6. For the analyses in this paper, were the exposure(s) of interest measured prior to the outcome(s) being measured?			
7. Was the timeframe sufficient so that one could reasonably expect to see an association between exposure and outcome if it existed?			
8. For exposures that can vary in amount or level, did the study examine different levels of the exposure as related to the outcome (e.g., categories of exposure, or exposure measured as continuous variable)?			
9. Were the exposure measures (independent variables) clearly defined, valid, reliable, and implemented consistently across all study participants?			
10. Was the exposure(s) assessed more than once over time?			
11. Were the outcome measures (dependent variables) clearly defined, valid, reliable, and implemented consistently across all study participants?			
12. Were the outcome assessors blinded to the exposure status of participants?			
13. Was loss to follow-up after baseline 20% or less?			
14. Were key potential confounding variables measured and adjusted statistically for their impact on the relationship between exposure(s) and outcome(s)?			

*CD, cannot determine; NA, not applicable; NR, not reported

**Quality Rating (Good, Fair, or Poor)**	
**Rater #1 initials:**	
**Rater #2 initials:**	
**Additional Comments (If POOR, please state why):**	

For peer review only

PRISMA-P (Preferred Reporting Items for Systematic review and Meta-Analysis Protocols) 2015 checklist: recommended items to address in a systematic review protocol*

Section and topic	Item No	Checklist item	(Page No.#)
**ADMINISTRATIVE INFORMATION**			
Title:			
Identification	1a	Identify the report as a protocol of a systematic review	1
Update	1b	If the protocol is for an update of a previous systematic review, identify as such	1
Registration	2	If registered, provide the name of the registry (such as PROSPERO) and registration number	2
Authors:			
Contact	3a	Provide name, institutional affiliation, e-mail address of all protocol authors; provide physical mailing address of corresponding author	1
Contributions	3b	Describe contributions of protocol authors and identify the guarantor of the review	8
Amendments	4	If the protocol represents an amendment of a previously completed or published protocol, identify as such and list changes; otherwise, state plan for documenting important protocol amendments	N/A
Support:			
Sources	5a	Indicate sources of financial or other support for the review	1
Sponsor	5b	Provide name for the review funder and/or sponsor	
Role of sponsor or funder	5c	Describe roles of funder(s), sponsor(s), and/or institution(s), if any, in developing the protocol	
**INTRODUCTION**			
Rationale	6	Describe the rationale for the review in the context of what is already known	3-4
Objectives	7	Provide an explicit statement of the question(s) the review will address with reference to participants, interventions, comparators, and outcomes (PICO)	4-5
**METHODS**			
Eligibility criteria	8	Specify the study characteristics (such as PICO, study design, setting, time frame) and report characteristics (such as years considered, language, publication status) to be used as criteria for eligibility for the review	4-5
Information sources	9	Describe all intended information sources (such as electronic databases, contact with study authors, trial registers or other grey literature sources) with planned dates of coverage	6
Search strategy	10	Present draft of search strategy to be used for at least one electronic database, including planned limits, such that it could be repeated	6 and Appendix-1

For peer review only

Study records:			
Data management	11a	Describe the mechanism(s) that will be used to manage records and data throughout the review	6-7
Selection process	11b	State the process that will be used for selecting studies (such as two independent reviewers) through each phase of the review (that is, screening, eligibility and inclusion in meta-analysis)	6 and Appendix 2
Data collection process	11c	Describe planned method of extracting data from reports (such as piloting forms, done independently, in duplicate), any processes for obtaining and confirming data from investigators	6-7 and Appendix 3
Data items	12	List and define all variables for which data will be sought (such as PICO items, funding sources), any pre-planned data assumptions and simplifications	6-7
Outcomes and prioritization	13	List and define all outcomes for which data will be sought, including prioritization of main and additional outcomes, with rationale	6-7
Risk of bias in individual studies	14	Describe anticipated methods for assessing risk of bias of individual studies, including whether this will be done at the outcome or study level, or both; state how this information will be used in data synthesis	7 and Appendix 4
Data synthesis	15a	Describe criteria under which study data will be quantitatively synthesised	6-7
	15b	If data are appropriate for quantitative synthesis, describe planned summary measures, methods of handling data and methods of combining data from studies, including any planned exploration of consistency (such as I^2, Kendall's τ)	N/A
	15c	Describe any proposed additional analyses (such as sensitivity or subgroup analyses, meta-regression)	7
	15d	If quantitative synthesis is not appropriate, describe the type of summary planned	7
Meta-bias(es)	16	Specify any planned assessment of meta-bias(es) (such as publication bias across studies, selective reporting within studies)	7
Confidence in cumulative evidence	17	Describe how the strength of the body of evidence will be assessed (such as GRADE)	7

*** It is strongly recommended that this checklist be read in conjunction with the PRISMA-P Explanation and Elaboration (cite when available) for important clarification on the items. Amendments to a review protocol should be tracked and dated. The copyright for PRISMA-P (including checklist) is held by the PRISMA-P Group and is distributed under a Creative Commons Attribution Licence 4.0.**

From: Shamseer L, Moher D, Clarke M, Ghersi D, Liberati A, Petticrew M, Shekelle P, Stewart L, PRISMA-P Group. Preferred reporting items for systematic review and meta-analysis protocols (PRISMA-P) 2015: elaboration and explanation. BMJ. 2015 Jan 2;349(jan02 1):g7647.

For peer review only
